# The UNU-CBGs: development and deployment of a real international open source Casemix grouper for resource challenged countries

**DOI:** 10.1186/1472-6963-11-S1-A4

**Published:** 2011-10-19

**Authors:** SM Aljunid, SM Hamzah, SA Mutalib, AM Nur, N Shafie, S Sulong

**Affiliations:** 1 UNU-IIGH, UNU-International Institute for Global Health, Kuala Lumpur, Federal Territory, 56000, Malaysia; 2 International Casemix and Clinical Coding Centre, Universiti Kebangsaan Malaysia, Kuala Lumpur, Federal Territory, 56000, Malaysia

## Introduction

Although the Casemix system has been in existence for more than three decades, the deployment of this system in developing countries is very erratic. A call by international donors to enhance the efficiency of existing social insurance schemes by introducing the prospective payment method has prompted many developing countries to use the Casemix system. Unfortunately, the lack of a low-cost, reliable and customizable Casemix grouper, based on an open source, is a major obstacle to the adoption of the Casemix system in these developing countries.

## Methods

UNU-IIGH (United Nations University International Institute for Global Health), in collaboration with the International Centre on Casemix and Clinical Coding of UKM (Universiti Kebangsaan Malaysia) and UNU-IIST (United Nations University International Institute for Software Technology), has developed a Casemix grouper targeted for use in developing countries. The UNU Casemix grouper is a universal, dynamic and advanced grouper employing ICD-10 for disease classification, and ICD-9CM for procedure classification. The grouper covers a wide range of healthcare services in primary, secondary and tertiary settings. These include ambulatory services, in-patient services, daycare surgery, chemotherapy, rehabilitation and mental health.

## Results

The UNU Casemix grouper is the first grouper that covers acute, sub-acute and chronic long-stay patients. The grouper extends beyond the classical DRG (Diagnosis Related Groups) classification system by taking into account not only diagnoses and procedures for the creation of ISO-resource groups, but also special prostheses, special investigations and high-cost drugs.

The grouper is structured around 32 CMG (Casemix Major Groups) and 1220 refined groups called CBGs (Case-Based Groups). For each of these CBGs, the severity and resource intensity level ranges from three to a maximum of eight. This is to provide greater flexibility and the more refined classification required when the system is used for provider payment and resource allocations.

Also, the grouper includes three additional software applications to facilitate the implementation of the Casemix system in developing countries. These are Data Tool Pro. Version 2.0 for data collection; CCM Version 2.0 for clinical costing; and Code Assist, which is a digital coding tool to aid coders in enhancing their productivity. Initially launched in 2009, this grouper is currently being deployed in four countries (Indonesia, the Philippines, Uruguay and Malaysia). Six other countries are in the planning stages of adopting the system.

## Conclusions

The development and deployment of the universal, dynamic and advanced UNU Casemix grouper has enabled more developing countries with limited financial resource to implement and sustain the use of a Casemix system and reap the long-term benefits of this health management tool.

